# ADAM9 Mediates Triple-Negative Breast Cancer Progression via AKT/NF-κB Pathway

**DOI:** 10.3389/fmed.2020.00214

**Published:** 2020-06-19

**Authors:** Rui Zhou, William C. S. Cho, Victor Ma, Wah Cheuk, Yik-Ka So, S. C. Cesar Wong, Mingrong Zhang, Cong Li, Yujie Sun, Hong Zhang, Lawrence W. C. Chan, Mei Tian

**Affiliations:** ^1^Department of Health Technology and Informatics, The Hong Kong Polytechnic University, Hong Kong, China; ^2^Department of Nuclear Medicine and Medical PET Center, The Second Hospital of Zhejiang University School of Medicine, Hangzhou, China; ^3^Department of Clinical Oncology, Queen Elizabeth Hospital, Hong Kong, China; ^4^Department of Pathology, Queen Elizabeth Hospital, Hong Kong, China; ^5^Department of Advanced Nuclear Medicine Sciences, National Institute of Radiological Sciences, Chiba, Japan; ^6^Key Laboratory of Smart Drug Delivery, Ministry of Education, School of Pharmacy, Fudan University, Shanghai, China; ^7^State Key Laboratory of Membrane Biology, Biomedical Pioneer Innovation Center, School of Life Sciences, Peking University, Beijing, China; ^8^Key Laboratory for Biomedical Engineering of Ministry of Education, Zhejiang University, Hangzhou, China; ^9^The College of Biomedical Engineering and Instrument Science of Zhejiang University, Hangzhou, China; ^10^Department of Nuclear Medicine, The First Hospital of Shanxi Medical University, Taiyuan, China

**Keywords:** A disintegrin and metalloprotease 9 (ADAM9), AKT, NF-κB, triple-negative breast cancer (TNBC), tumor progression

## Abstract

Upregulation of a disintegrin and metalloprotease 9 (ADAM9) is correlated with progression of cancers, such as prostate, bladder, and pancreatic cancers. However, its role in triple-negative breast cancer (TNBC) is still unclear. Our study aimed to investigate whether ADAM9 is upregulated and promoted the aggressiveness in TNBC. Breast cancer cell lines and patient specimens were used to evaluate the ADAM9 expression by western blotting and immunohistochemistry staining, respectively. Compared with the non-TNBC, ADAM9 expression was significantly increased in TNBC cells and TNBC patient specimens. Based on the data acquired from public databases, the correlation between ADAM9 expression and breast cancer patient survival was analyzed by Kaplan-Meier method. It was shown that ADAM9 overexpression was significantly correlated with poorer survival in patients with TNBC. Furthermore, ADAM9 in TNBC cells was knocked down by small interference RNA and then studied by the MTT/colony formation assay, wound healing assay and transwell invasion assay on the cell proliferation, migration, and invasion, respectively. We found that inhibiting ADAM9 expression suppressed TNBC cell proliferation, migration, and invasion by lowering the activation of AKT/NF-κB pathway. Our results demonstrated that ADAM9 is an important molecule in mediating TNBC aggressiveness and may be a potential useful therapeutic target in TNBC treatment.

## Introduction

Triple-negative breast cancer (TNBC) is defined as absence of estrogen receptor (ER), progesterone receptor (PR), and human epidermal growth factor receptor 2 (HER2) expression. Compared with the non-TNBC, patients with TNBC typically have a more aggressive clinical phenotype, poorer prognosis, and limited benefit from hormonal or anti-HER2 therapy (1). Targeted therapies, including epidermal growth factor receptor (EGFR) inhibitors (Cetuximab), poly (ADP-ribose) polymerase (PARP) inhibitors, and phosphatidylinositol 3-kinase (PI3K) inhibitors, are approved to be used in TNBC clinical trials, however, no significant improvement have been achieved in phase 3 trials (2, 3).

Alternatively, a disintegrin and metalloproteinases (ADAMs), a family of transmembrane proteins with disintegrin and metalloproteinase domain, were found to be a promising target for cancer treatment (4). To date, 21 members of the family have been identified in humans, of which 13 have functional protease activities to release ligands to stimulate cell proliferation and migration (5). Particularly, ADAM9, as a member of ADAMs that has functional protease activities, involves in cell-cell interactions and extracellular matrix degradation by binding with integrins including α6β1 and αvβ5 (6, 7). ADAM9 is firstly synthesized as a precursor of the enzyme whose molecular weight is about 110 kDa. Then, ADAM9 precursor is cleaved at a consensus cleavage sequence that is located between pro- and metalloprotease domain by the pro-protein convertase furin in the medial-Golgi apparatus, and becomes the mature ADAM9 (84 kDa) as a membrane-anchored protein with protease activity (8). Previous studies indicated that ADAM9 mediates cancer progression via regulating epithelial-to-mesenchymal transition (EMT) (9), shedding EGFR ligands (10), and activating EGFR/AKT signaling pathway (11). Overexpressed ADAM9 was found in cancers including esophageal squamous cell carcinoma (11), pancreatic cancer (12), prostate cancer (13), and breast cancer (14). However, whether ADAM9 is upregulated and contributes to the aggressiveness in TNBC still remains unclear.

To better understand the role of ADAM9 in TNBC progression, we investigated ADAM9 expression in clinical samples, analyzed the correlation between ADAM9 expression and survival of patients with TNBC, and examined the cancer cell motility and invasiveness by knocking down the expression of ADAM9 in human TNBC cell lines, MDA-MB-231 and Hs578t, using small interference RNA (siRNA). We showed that ADAM9 expression is upregulated in TNBC while suppressed ADAM9 expression inhibited the proliferation, migration, and invasion of TNBC cells via AKT/NF-κB pathway.

## Materials and Methods

### Cell Lines and Patient Specimens

MDA-MB-231 (TNBC cell line), Hs578t (TNBC cell line), MDA-MB-361 (ER+ and HER2+), and MCF-7 (ER+ and PR+) were cultured in DMEM medium (Thermo Fisher Scientific, Maryland, USA) with 10% fetal bovine serum (FBS) (Gibco, Maryland, USA). SKBR3 (HER2+) was cultured in McCoy's 5A medium (Thermo Fisher Scientific) with 10% FBS (Gibco). T47D (ER+ and PR+) was cultured in RPMI 1640 medium (BasalMedia, Shanghai, China) with 10% FBS. MCF-10A (Non-tumorigenic epithelial cell line) was cultured in MEBM basal medium (Lonza, Maryland, USA) supplemented with 100 ng/mL cholera toxin (Sigma, Missouri, USA), insulin, hEGF, bovine pituitary extract (BPE) and hydrocortisone (Lonza).

MDA-MB-231, Hs578t, T47D, MCF-7, and MCF-10A were purchased from China Center for Type Culture Collection. MDA-MB-361 was purchased from The American Type Culture Collection (ATCC). SKBR3 was a gift from Prof. Benjamin Y. M. Yung (The Hong Kong Polytechnic University, HKSAR, China).

Breast tumor tissues were obtained from 44 patients, and patient specimens were divided into 2 groups including TNBC group (*n* = 24) and non-TNBC group (*n* = 20). These patient specimens were obtained from the Queen Elizabeth Hospital, HKSAR, China. The study was approved by the Research Ethics Committee of the Kowloon Central / Kowloon East Cluster under the Hospital Authority (Ref: KC/KE-19-0114/ER-2).

### Immunohistochemistry (IHC) Staining

Sections (5 μm) were cut from formalin-fixed paraffin-embedded specimens and mounted on Superfrost slides (Menzel Glaser, Lower Saxony, Germany) and gradually rehydrated after deparaffinization by xylene. Antigen retrieval was achieved by super heating in microwave oven with pH 6 citrate buffer for 15 min after boiling. The primary antibody ADAM9 (R&D System, Minnesota, USA) was diluted to 1:50 in Antibody Diluent (Dako, Denmark). After incubation at room temperature for 30 min, antigen-antibody reaction was detected by using anti-goat HRP-DAB tissue staining kit (R&D System). The slides were then counterstained with haematoxylin. For the negative control, the primary antibody was omitted. The immunohistochemistry staining results were evaluated by two experienced pathologists. The ADAM9 IHC intensity was classified into 5 grades (0 = negative, 1 = weak, 2 = moderate, 3 = strong, and 4 = prominent staining). The ADAM9 staining percentage was calculated by quantifying stained cells. ADAM9 expression level was calculated by applying the following formula: Expression level = ADAM9 staining intensity x ADAM9 staining percentage.

### SiRNA Transfection and Protein Inhibition

MDA-MB-231, Hs578t, and MCF-7 cells (3 × 10^5^ cells/well) were cultured at 5% CO_2_ and 37°C for 24 h in a 6-well plate, and transfected with ADAM9 siRNA (RiboBio, Guangdong, China) by Lipofectamine 2000 (Thermo Fisher Scientific). After 6 h, the completed medium was replaced by conditioned medium (DMEM). Cells transfected with scrambled siRNA was regarded as negative control (NC), and the blank control (BC) was defined as cancer cells only treated with Lipofectamine 2000. Scrambled siRNA with green fluorescence protein (GFP, RiboBio) was transfected in MDA-MB-231 cells for 3 and 6 h to investigate the transfection efficiency.

GSK690693 (Selleckchem) and MK2206-2HCl (Selleckchem) are pan-AKT inhibitor and p-AKT inhibitor at Ser473 and Thr308, respectively. The expression of AKT and p-AKT in MDA-MB-231 cells was inhibited by GSK690693 and MK2206-2HCl to investigate whether AKT could regulate the phosphorylation of NF-κB in MDA-MB-231 cells.

### Western Blotting and Real-Time Quantitative PCR (RT-qPCR)

Western blotting was performed as described previously (15). All the types of cells were lysed by RIPA buffer with cocktail protease and phosphatase inhibitors (Thermo Fisher Scientific). Total protein extracts (40 μg) were subjected to western blotting analysis with antibodies against the following proteins: ADAM9 (R&D system); p-EGFR (Tyr1068), EGFR, p-MAPK (Tyr202/204), MAPK, GAPDH, p-AKT (Ser473), AKT, p-IKKα/β (Ser176/180), IKKβ, p-NF-κBp65 (Ser536), NF-κBp65, p-IκBα (Ser32), IκBα, and β-actin (Cell Signaling, Massachusetts, USA).

Total RNA was extracted from all the types of cells by using TRIzol Reagent (Thermo Fisher Scientific) according to the vendor's instruction. The cDNA synthesis was achieved by using PrimeScript reverse transcription reagent Kit with gDNA Eraser (TaKaRa, Shiga, Japan). Quantitative PCR was performed with ADAM9 and GAPDH primers by using SYBR Green PCR Master Mix (Roche, Baden-Württemberg, Germany) in a real-time PCR System (Applied Biosystems 7500, Thermo Fisher Scientific). Primer sequences were as follows: ADAM9, 5′-CCTCGGGGACCCTTCGTGT-3′ and 5′-ATCCCATAACTCGCATTCTCTAAA-3′; GAPDH, 5′-CCACCCATGGCAAATTCCATGGCA-3′ and 5′-TCTAGACGGCAGGTCAGGTCCACC-3′. Quantitative analysis of RT-qPCR was achieved by the 2^−ΔΔ^Ct method.

### Cell Growth Assay

MDA-MB-231, Hs578t, and MCF-7 cells were transfected with ADAM9 siRNA, NC and BC were seeded in 96-well plate for 24 h. After 1, 2, 3, or 4 days of transfection, 3-(4,5-dimethylthiazol-2-yl)-2,5-diphenyltetrazolium bromide (MTT) assay was performed by using Cell Proliferation Kit I (Roche). Briefly, the MTT (10 μL) was added into the medium. After 4 h incubation, the solubilization solution (100 μL) was added into the medium following overnight incubation at 37°C and 5% CO_2_. The absorbance at 560 nm was measured by a microplate reader (Bio-Rad, California, USA). For colony formation assay, MDA-MB-231 and Hs578t cells transfected with ADAM9 siRNA, NC and BC (500 cells/well) were seeded in six-well plate and incubated in DMEM medium with 10% FBS (completed medium) for 12 days. Colonies were fixed with 4% PFA for 15 min, stained with 0.1% crystal violet for 30 min and recorded by a digital camera (Nikon, Tokyo, Japan).

### Wound Healing and Transwell Invasion Assay

For the wound healing assay, briefly, cells transfected with ADAM9 siRNA, NC and BC (3 × 10^5^ cells/mL) were seeded into two inserted wells of the Culture-Insert 2 Well (ibidi, Wisconsin, USA) and incubated with completed medium. After the inserted wells removed, the cell-free gap generated by two inserted wells was observed and photographed by using a light microscope every 6 h for 24 h. Matrigel-coated Transwell invasion assay (Cell Biolabs CytoSelectTM Cell Invasion Assay Kit) was performed according to the vendor's protocol. Cells transfected with ADAM9 siRNA, NC and BC (2x10^5^ cells/mL) were added into the upper chamber contained serum-free medium (DMEM containing 0.5% BSA, 2 mM CaCl_2_ and 2 mM MgCl_2_). Completed medium was added into the lower chamber of the invasion plate. After 24 h incubation at 37°C in 5% CO_2_, invasive cells in the lower chamber were stained with cell stain solution and observed for at least three individual fields per insert by a light microscope (Nikon).

### Enzyme-Linked Immunosorbent Assay (ELISA)

To investigate whether ADAM9 could shed HB-EGF to activate EGFR/AKT pathway in MDA-MB-231 cells, ELISA was performed to assess the HB-EGF protein level in supernatant of MDA-MB-231 cells culture medium. HB-EGF level in culture supernatants was measured by using HB-EGF ELISA kit (R&D Systems) according to the manufacture's recommendation.

### Survival Analysis

The correlation between ADAM9 expression and survival of patients [relapse free survival (RFS), overall survival (OS), and distant metastasis free survival (DMFS)] was investigated by using Kaplan-Meier (KM) plotter integrative data analysis tool (16) (http://kmplot.com/analysis/). Briefly, the gene symbol was set as ADAM9 (202381_at) and patients were split by median. Probe set options were chosen as “the user selected probe set.” All the other settings (intrinsic subtype, following up threshold, lymph node status, TP53 status, pietenpol subtype, medication that patients received, and included dataset) were set as the default value.

### Statistical Analysis

All data were presented as the mean ± S.D. One-way ANOVA analysis and Student's *t*-test were performed to compute significance of difference. Non-parametric test (Mann-Whitney *U*-test) was used to compare ADAM9 mRNA expression between TNBC samples (*n* = 34) and ER+ breast cancer samples (*n* = 37), as well as ADAM9 mRNA expression between TNBC cell lines (*n* = 14) and non-TNBC cell lines (*n* = 11) obtained from Gene Expression Omnibus (GEO, https://www.ncbi.nlm.nih.gov/geo/, GSE58135) database (17). IHC results were also analyzed by using Mann-Whitney U test. ADAM9 mRNA expression in 22 TNBC cell lines and 28 non-TNBC cell lines was obtained from Cancer Cell Line Encyclopedia (CCLE) (http://portals.broadinstitute.org/ccle). ADAM9 expression in hTERT-HME1, MCF-7, SKBR3, and T47D was obtained from The Human Protein Atlas (http://www.proteinatlas.org). *P* < 0.05 was considered as statistical significance.

Spearman correlation analysis was performed using PASW Statistics 18. Gene expression data (version: HiseqV12) and Reverse Phase Protein Array Replicates-Based-Normalized (RPPA-RBN) data (version: 2018-06-13) of The Cancer Genome Atlas (TCGA) BRCA were obtained from National Cancer Institute GDC Data Portal (https://portal.gdc.cancer.gov/) and UCSC Cancer Genome Browser (http://genome-cancer.ucsc.edu), respectively.

## Results

### Upregulation of ADAM9 in TNBC Primary Tumor and TNBC Cell Lines

**Figure 1:**
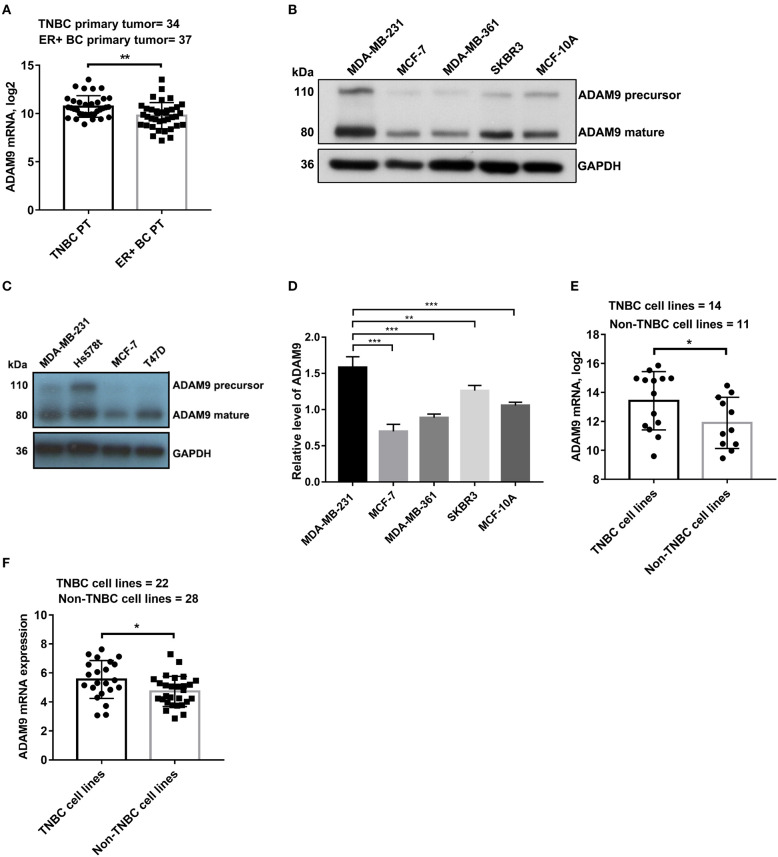
ADAM9 expression in breast clinical samples and breast cell lines. **(A)** ADAM9 was markedly upregulated in clinical samples of TNBC primary tumor (*n* = 34) compared to ER+ breast cancer primary tumor (*n* = 37) in NCBI GEO (GSE58135) database (PT = primary tumor); **(B)** ADAM9 protein expression was upregulated in MDA-MB-231 cells compared to the other non-TNBC cell lines (MCF-7, MDA-MB-361, SKBR3, and MCF-10A); **(C)** Western blotting showed the increased ADAM9 protein expression in MDA-MB-231 and Hs578t cell lines; **(D)** RT-qPCR analysis indicated upregulated ADAM9 mRNA expression in TNBC cell line; **(E)** TNBC cell lines showed significantly overexpressed ADAM9 mRNA compared with non-TNBC cell lines in GSE58135 dataset; **(F)** ADAM9 mRNA expression from CCLE showed overexpressed ADAM9 in TNBC cell lines compared with non-TNBC cell lines. (*p-value < 0.05, **p-value < 0.01, ***p-value < 0.001).

To determine whether ADAM9 is overexpressed in TNBC primary tumor when compared to non-TNBC primary tumor, we investigated the ADAM9 expression in breast primary tumor and breast cancer cell lines. Significant upregulation of ADAM9 mRNA was observed in TNBC primary tumor compared to ER+ breast cancer primary tumor from published GEO datasets (GSE58135) (Figure 1A). Similarly, markedly increased ADAM9 protein (Figure 1B) was found in MDA-MB-231 cells compared to the other breast cell lines (MCF-7, MDA-MB-361, SKBR3, and MCF-10A). To further support our findings, Hs578t cells, another TNBC cell line, was used to investigate the ADAM9 expression. The western blot result showed the same pattern with MDA-MB-231 cells (Figure 1C). RT-qPCR result indicated that gene expression level of ADAM9 in MDA-MB-231 was higher than non-TNBC cell lines (Figure 1D). Furthermore, from the published tumor dataset (GSE58135), we also found that ADAM9 mRNA expression was also significantly increased in TNBC cell lines (MDA-MB-468, HCC-38, SUM-159, HCC-1599, MDA-MB-436, BT-549, MDA-MB-231, HCC-70, HCC-1143, HCC-1937, SUM-149, MDA-MB-157, BT-20, and SUM-102) compared with non-TNBC cell lines (ZR-75-1, BT-474, MCF-7, T-47-D, SKBR3, MDA-MB-134, MDA-MB-361, HCC-1954, ZR-75-30, MDA-MB-453, and HCC-1569) (Figure 1E). ADAM9 expression of breast cancer cell lines from CCLE showed the same pattern in TNBC cell lines when compared with non-TNBC cell lines (Figure 1F).

### Overexpressed ADAM9 in TNBC Patient Specimens

**Figure 2:**
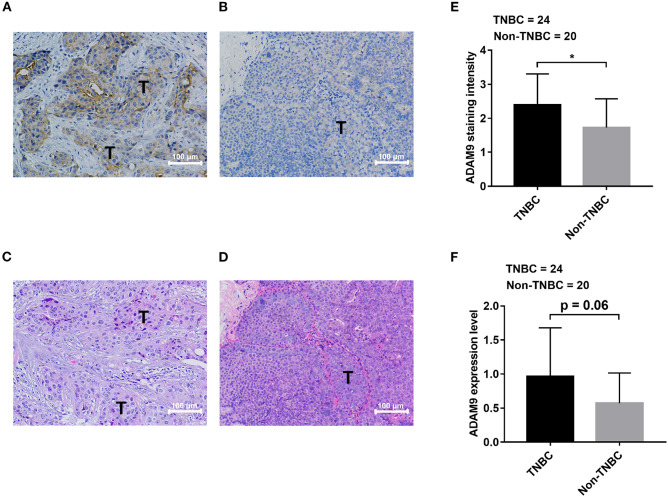
ADAM9 expression is upregulated in tissues from TNBC patients compared with that of from non-TNBC patients. **(A)** Abundant staining of ADAM9 in tumor tissue from TNBC patients (*n* = 24); **(B)** Relatively weak staining of ADAM9 in tumor tissue from non-TNBC patients (*n* = 20); **(C,D)** Hematoxylin-eosin staining images of TNBC tumor tissues **(C)** and non-TNBC tumor tissues **(D)**; **(E,F)** Independent sample *t*-test showed that significantly enhanced ADAM9 staining intensity **(E)** and expression level **(F)** in TNBC group (*n* = 24) compared with non-TNBC group (*n* = 20). (Expression level = ADAM9 staining intensity x ADAM9 staining percentage; **A–D**. X200 magnification; scale bars = 100 μm; *p-value < 0.05.

We retrieved breast cancer specimens from clinic and investigated their ADAM9 protein expression. Overall, 44 specimens of breast cancer patients were obtained, at which 24 of them are TNBC specimens and 20 specimens are non-TNBC. The clinical information and IHC results of patients were summarized in Table 1. We observed abundant staining of ADAM9 in tumor tissues from TNBC patients (Figure 2A) compared with tumor tissues from non-TNBC patients (Figure 2B). Hematoxylin-eosin staining was used to indicate the tumor cells site in TNBC tissues (Figure 2C) and non-TNBC tissues (Figure 2D). Statistical analysis showed that ADAM9 staining intensity (Figure 2E) was significantly increased in TNBC group compared with that of in non-TNBC group. Though no significant increase of ADAM9 expression level was found in TNBC group, we found that there was an increase trend of ADAM9 expression level in TNBC compared with non-TNBC group (Figure 2F, *p* = 0.06).

**Table 1:** Immunohistochemistry staining for patients with breast cancer.

**Case number**	**Diagnosis**	**Histology**	**ER**	**PR**	**HER2**	**ADAM9 IHC intensity**	**ADAM9 staining percentage**
1	TNBC, metastasis	IDC	/	/	/	3+	70%
2	TNBC, metastasis	IDC	/	/	/	1+	30%
3	TNBC, metastasis	IDC	/	/	/	3+	40%
4	TNBC, metastasis	IDC	/	/	/	3+	50%
5	TNBC, metastasis	IDC	/	/	/	2+	40%
6	TNBC, metastasis	IDC	/	/	/	1+	70%
7	TNBC, LN metastasis	IDC	/	/	/	2+	20%
8	TNBC, LN metastasis	IDC	/	/	/	2.5+	20%
9	TNBC, LN metastasis	IDC	/	/	/	1+	60%
10	TNBC, LN metastasis	IDC	/	/	/	3+	30%
11	TNBC, LN metastasis	IDC	/	/	/	2+	10%
12	TNBC, LN metastasis	IDC	/	/	/	3.5+	70%
13	TNBC, non-metastasis	IDC	/	/	/	1+	20%
14	TNBC, non-metastasis	IDC	/	/	/	2+	60%
15	TNBC, non-metastasis	IDC	/	/	/	1+	10%
16	TNBC, non-metastasis	IDC	/	/	/	3+	60%
17	TNBC, non-metastasis	IDC	/	/	/	2.5+	30%
18	TNBC, non-metastasis	IDC	/	/	/	4+	30%
19	TNBC, non-metastasis	IDC	/	/	/	2+	40%
20	TNBC, non-metastasis	IDC	/	/	/	2.5+	20%
21	TNBC, non-metastasis	IDC	/	/	/	3+	60%
22	TNBC, non-metastasis	IDC	/	/	/	3.5+	70%
23	TNBC, non-metastasis	IDC	/	/	/	2.5+	10%
24	TNBC, non-metastasis	IDC	/	/	/	3.5+	10%
25	Non-TNBC	IDC	6	8	0	1+	50%
26	Non-TNBC	IDC	8	8	1	0.5+	10%
27	Non-TNBC	IDC	7	5	2	2+	20%
28	Non-TNBC	IDC	8	8	2	1+	70%
29	Non-TNBC	IDC	7	0	3	1+	70%
30	Non-TNBC	IDC	7	6	2	0.5+	10%
31	Non-TNBC	IDC	8	8	2	2+	25%
32	Non-TNBC	IDC	8	5	2	2+	10%
33	Non-TNBC	IDC	8	8	1	0.5+	40%
34	Non-TNBC	IDC	8	8	0	0.5+	50%
35	Non-TNBC	IDC	8	6	1	2+	30%
36	Non-TNBC	IDC	8	7	2	2+	40%
37	Non-TNBC	IDC	8	8	0	3+	40%
38	Non-TNBC	IDC	8	8	2	3+	60%
39	Non-TNBC	IDC	6	8	1	2.5+	40%
40	Non-TNBC	IDC	8	8	2	2+	50%
41	Non-TNBC	IDC	8	0	2	2+	10%
42	Non-TNBC	IDC	8	0	2	3+	10%
43	Non-TNBC	IDC	8	7	3	2+	40%
44	Non-TNBC	IDC	8	7	2	2+	10%

*LN, lymph node; IDC, invasive ductal carcinoma; IHC, immunohistochemistry; TNBC, triple-negative breast cancer*.

### Correlations Between ADAM9 Expression and Survival of Patients

**Figure 3:**
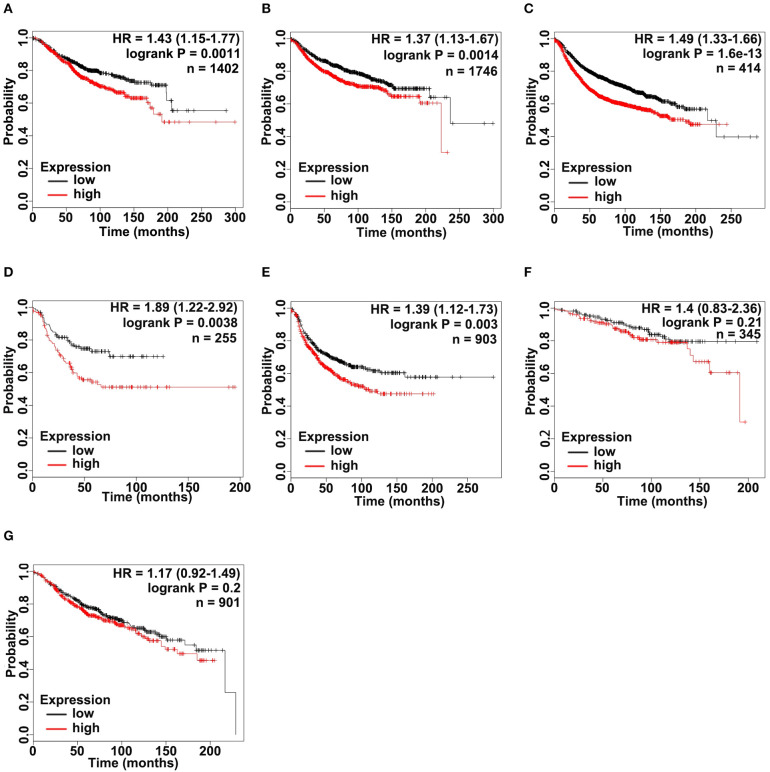
ADAM9 expression significantly correlates with poor survival in patients with breast cancer. **(A–C)** Kaplan-Meier (KM) plotter survival analysis tool (http://www.kmplot.com) indicated that ADAM9 expression was negatively correlated with OS (*n* = 1,402), DMFS (*n* = 1,746), and RFS (*n* = 414) of breast cancer patients; **(D)** High ADAM9 expression indicated the poor RFS (*n* = 255) of TNBC patients; **(E)** RFS of breast cancer patients with grade III tumors (*n* = 903); **(F)** RFS of breast cancer patients with grade I tumors (*n* = 345); **(G)** RFS of breast cancer patients with grade II tumors (*n* = 901). Patient samples were classified into high (red) and low (black) ADAM9 median expression group. Hazard ratio (HR) with 95% confidence interval and logrank *p*-value are displayed.

According to the upregulated ADAM9 expression in TNBC cell lines and tumors, we investigated whether upregulated ADAM9 expression is associated with poor prognosis of breast cancer patients, especially in patients of TNBC. Overall survival (OS), relapse free survival (RFS), and distant metastasis free survival (DMFS) were firstly investigated in all breast cancer patients included in Kaplan-Meier Plotter (http://www.kmplot.com). The results indicated that high ADAM9 mRNA expression was significantly correlated with poor OS (Figure 3A, *p* < 0.01), DMFS (Figure 3B, *p* < 0.01), and RFS (Figure 3C, *p* < 0.001) in breast cancer patients. Furthermore, the correlation between ADAM9 expression and lifespan was analyzed in TNBC patients, while limited cases of TNBC patients only allowed to conduct RFS analysis. Overexpressed ADAM9 mRNA level indicated reduced RFS in patients with TNBC (Figure 3D, *p* < 0.01). To further illustrate the potential role of ADMA9 in breast cancer progression, RFS analysis was performed in different grades of breast cancer patients. Increasing hazard ratio was found with increasing tumor grades, which indicated the important role of ADAM9 in high grade of breast cancer. Significant correlation was observed between upregulation of ADAM9 mRNA and poor RFS in patients with grade III tumor (Figure 3E, *p* < 0.01), while no significant correlation was found in patients with grade I and II tumors (Figure 3F, *p* = 0.21 and Figure 3G, *p* = 0.2).

### Inhibited Cell Proliferation, Migration, and Invasion in TNBC Cell Lines After ADAM9 Silencing

**Figure 4:**
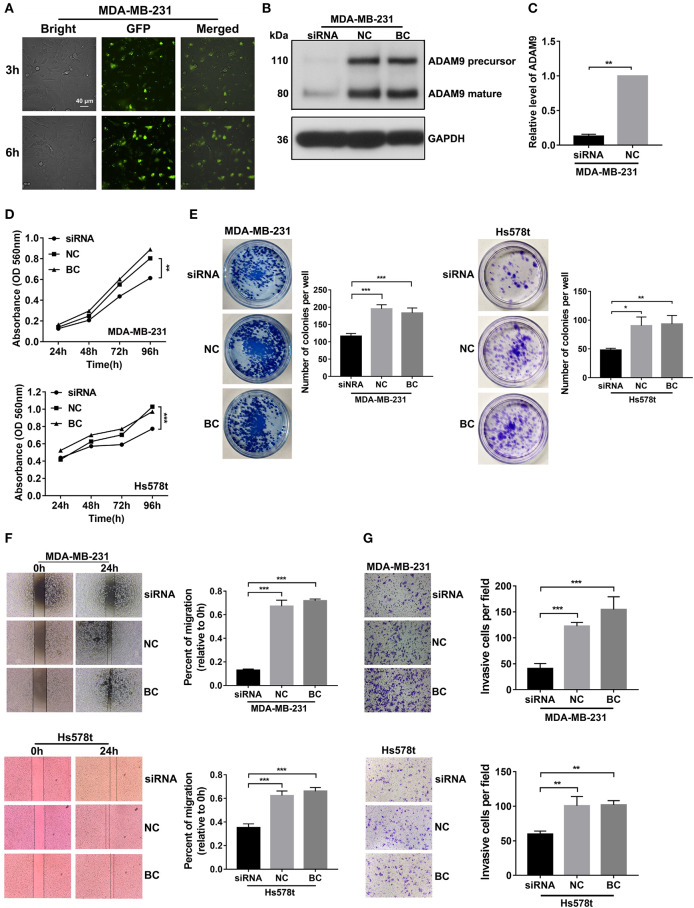
ADAM9 silencing in TNBC cells inhibits cell growth, migration, and invasion *in vitro*. **(A)** Scrambled siRNA had a satisficed transfection efficiency in MDA-MB-231 cells at 3 and 6 h by Lipofectamine 2000. (scale bars = 40 μm); **(B)** ADAM9 protein expression in MDA-MB-231 cells was markedly inhibited by ADAM9 siRNA; **(C)** RT-qPCR analysis indicated that ADAM9 mRNA was significantly suppressed by ADAM9 siRNA; **(D)** Inhibited cell proliferation was observed in MDA-MB-231 (top panel) and Hs578t (bottom panel) cells transfected with ADAM9 siRNA by the MTT assay; **(E)** Representative images indicated the inhibited colony formation in MDA-MB-231 (left panel) and Hs578t (right panel) cells transfected with ADMA9 siRNA compared to the NC and BC. Mean colonies were counted in six-well plate for each group. Bars = mean ± S.D., *n* = 3; **(F)** Suppressed cell migration in siRNA group were observed in MDA-MB-231 (top panel) and Hs578t cells (bottom panel) by wound healing assay; **(G)** ADAM9 silencing inhibited MDA-MB-231 (top panel) and Hs578t (bottom panel) cells invasion. (*p-value < 0.05, **p-value < 0.01, ***p-value < 0.001).

In attempt to find the role of ADAM9 in malignant behaviors of TNBC cells, we knocked down ADAM9 expression in TNBC cells by using siRNA. The transfection efficiency of Lipofectamine 2000 was investigated by using scrambled siRNA with GFP. The inflorescence image showed that scrambled siRNA with GFP was successfully transfected in MDA-MB-231 cells by Lipofectamine 2000 at 3 and 6 h (Figure 4A). Furthermore, compared with the NC, ADAM9 protein and mRNA expression were inhibited in MDA-MB-231 cells transfected with ADAM9 siRNA (Figures 4B,C). MTT assays showed that ADAM9 silencing inhibited proliferation of MDA-MB-231 and Hs578t cells (Figure 4D), while no such effect was observed on MCF-7 cells proliferation (Supplementary Figure 1A). Colony formation assay revealed that inhibited ADAM9 expression in MDA-MB-231 and Hs578t cells reduced the colonies compared with the control group (Figure 4E). The role of ADAM9 in cell migration and invasion was evaluated by wound healing assay and Matrigel invasion assay, respectively. Downregulated ADAM9 expression in MDA-MB-231 and Hs578t cells significantly reduced cells migration (Figure 4F) and invasion (Figure 4G). However, MCF-7 cell migration was not changed after ADAM9 silencing (Supplementary Figure 1B). Overall, decreased ADAM9 could suppress the proliferation, migration, and invasion of TNBC cells.

### ADAM9 Is Involved in AKT/NF-κB Pathway in TNBC Cells

**Figure 5:**
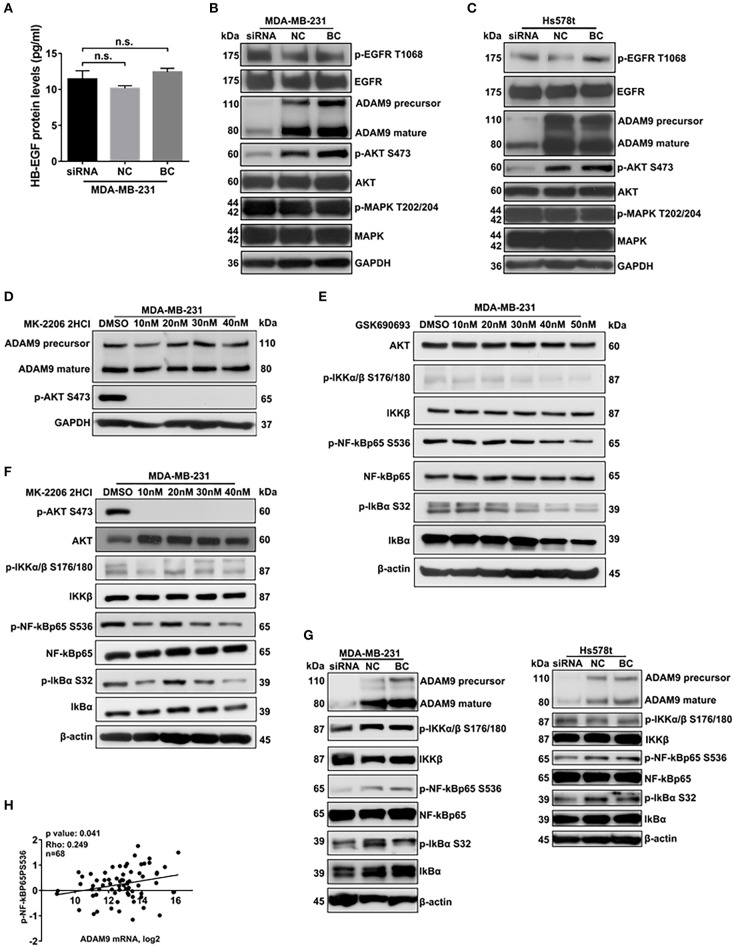
ADAM9 is involved in AKT/NF-κB pathway in TNBC cells. **(A)** No change of HB-EGF protein levels was detected in supernatant of MDA-MB-231 cells transfected with ADAM9 siRNA, NC and BC by HB-EGF ELISA assay; **(B,C)** Down-regulated ADAM9 expression inhibited the phosphorylation of AKT in MDA-MB-231 **(B)** and Hs578t cells **(C)**; **(D)** Specific p-AKT inhibitor, MK-2206 2HCl, significantly Inhibited phosphorylation of AKT without influencing the ADAM9 expression; **(E,F)** AKT (GSK690693) inhibitor **(E)** and p-AKT (MK-2206 2HCl) inhibitor **(F)** suppressed the phosphorylation of NF-κBp65 and IκBα, respectively; **(G)** Phosphorylation of NF-κBp65 and IκBα were inhibited as down-regulated ADAM9 expression in MDA-MB-231 (left panel) and Hs578t cells (right panel); **(H)** ADAM9 mRNA levels were significantly correlated with phosphorylation status of NF-κBp65 in Ser536 in TNBC patients from TCGA database (*n* = 68).

Previous studies indicated that ADAM9 activated EGFR-AKT pathway via shedding HB-EGF from cell surface to promote cancer progression (10). To investigate whether ADAM9 could shed HB-EGF to activate EGFR-AKT pathway in MDA-MB-231 cells, ELISA was performed to evaluate the HB-EGF protein levels in supernatant of MDA-MB-231 cells culture medium. Compared with the NC and BC, HB-EGF protein levels remained no change in supernatant of MDA-MB-231 cells transfected with ADAM9 siRNA (Figure 5A). Furthermore, the phosphorylation levels of EGFR and MAPK remained no significant changes in MDA-MB-231 and Hs578t cells after ADAM9 silencing (Figures 5B,C). However, the phosphorylation level of AKT was significantly reduced when ADAM9 expression was suppressed (Figures 5B,C). Meanwhile, inhibited p-AKT level had no significant effect on ADAM9 protein expression in MDA-MB-231 cells, which indicated that the phosphorylation of AKT is regulated by ADAM9 (Figure 5D). Previous studies indicated that activation of AKT could induce the phosphorylation of NF-κB (18). Therefore, we used AKT and p-AKT inhibitors to investigate whether AKT could regulate the phosphorylation of NF-κB in MDA-MB-231 cells. Suppressed AKT expression by GSK690693 in MDA-MB-231 cells indicated that p-IKKα/β, p-NF-κBp65, and p-IkBα levels were reduced (Figure 5E). Additionally, MK-2206 2HCl demonstrated that reduced p-AKT level could lead to the decrease of p-IKKα/β, p-NF-κBp65, and p-IκBα (Figure 5F). The use of GSK690693 and MK-2206 2HCl revealed that AKT could regulate the activation of NF-κB complex in MDA-MB-231 cells. After ADAM9 expression was pronouncedly suppressed in MDA-MB-231 (Figure 5G, left panel) and Hs578t cells (Figure 5G, right panel), the p-NF-κBp65 and p-IκBα levels were significantly reduced. Besides, significant correlation between ADAM9 mRNA expression level and NF-κBp65 activity (phosphorylation on serine 536) was observed in TNBC samples (*n* = 68) from TCGA database (Figure 5H). In particular, the ADAM9 mRNA level were positively correlated with matrix metalloproteinase 9 (MMP9) (Supplementary Figure 2A) and MMP2 mRNA levels (Supplementary Figure 2B).

## Discussion

Several members of ADAMs, such as ADAM10, ADAM12, and ADAM15, have been reported to be overexpressed in human cancers (19–21). ADAMs facilitate cancer progression mostly via their proteolytic activity. ADAM17 promoted cancer progression mainly by shedding Tumor Necrosis Factor-alpha (TNF-α) to activate the downstream signaling pathways (22). HER ligands can also be released by ADAMs (ADAM10 and ADAM12) to promote cancer cell growth, migration, and invasion (23, 24). Besides, several members of ADAMs were reported to involve in epithelial-mesenchymal transition (EMT) processes in cancer. ADAM12 is participated in TGF-β mediated EMT in breast cancer, while ADAM17 induces EMT in colorectal cancer via Notch signaling pathway (25, 26). In this study, we found that ADAM9 expression is upregulated in TNBC cells and patients compared with the non-TNBC. Enhanced ADAM9 expression is related to poorer survival in patients with TNBC. To our knowledge, this study is the first report that ADAM9 overexpression promotes the cell proliferation, migration, and invasion in TNBC cells by activating the AKT/NF-κB pathway.

ADAM9 expression was increased in tumor tissues from TNBC patients and TNBC cells compared with the non-TNBC tumor tissues and cell lines, which is consistent with the previous finding that ADAM9 was overexpressed and associated with a more aggressive phenotype of breast cancer (26). Interestingly, MCF-10A (a normal epithelial breast cell line) expresses relatively high ADAM9 expression than non-TNBC cell lines, which might be caused by the culture method of immortalized MCF-10A cell line. The recommended culture medium by ATCC is Mammary Epithelial Cell Growth Medium (MEGM) BulletKit, which adds hEGF as a supplement. MCF-10A cells have a strong migratory response to EGF. In this condition, MCF-10A might have a higher ADAM9 expression than some breast cancer cell lines as a response to additional EGF in cultural medium. HTERT-HME1, another immortalized human breast epithelial cell line cultured with MEGM BulletKit, also has higher ADAM9 expression (normalized expression = 35.2) than some other breast cancer cell lines (MCF-7 = 4, SKBR3 = 17.9 and T47D = 14.5), which is similar with MCF-10A. Though MCF-10A has a relatively high ADAM9 expression, it still behaves as a non-tumorigenic epithelial cell line. Survival analysis indicated that ADAM9 overexpression was correlated with poorer outcome in patients with breast cancer and TNBC. In agreement with this result, increased ADAM9 expression was tightly associated with poorer prostate-specific antigen RFS in prostate cancer (27). Upregulation of ADAM9 expression was found to be correlated with poorer patient survival in renal cell cancer (13). Furthermore, higher ADAM9 expression correlated with poorer RFS in higher-grade breast cancer in our study. This correlation was also observed in pancreatic ductal adenocarcinomas and renal cell cancer that higher ADAM9 expression is related to poorer tumor differentiation and patient's prognosis (12, 13). These findings supported the hypothesis that higher ADAM9 expression in tumors predicts a more aggressive phenotype and poorer clinical outcome (28).

Previously, enhanced ADAM9 expression has been shown to promote cancer progression by increasing cancer cells motility (29), invasion (30), and proliferation (31). We demonstrated that suppressed ADAM9 expression attenuated the cell proliferation, migration, and invasion in TNBC cells. But, Fry and Toker indicated that soluble (ADAM9-S) and transmembrane (ADAM9-L) silencing induced the migration of BT549 breast cancer cells (32). Furthermore, they found that overexpressed ADAM9-S promoted migration in BT549, while ADAM9-L inhibited migration. Whether ADAM9-S and ADAM9-L are existed in MDA-MB-231 and Hs578t cells is needed to be further investigated. ADAM9 was reported to facilitate the cancer progression by mediating ectodomain shedding of HB-EGF and major histocompatibility complex class I-related chain A (MICA) (10, 33). Increased HB-EGF level in supernatants and decreased MICA level on cancer cells surface activated the EGFR pathway and induces immune escape, respectively (11, 34). However, our results indicated that HB-EGF, p-EGFR, and p-MAPK level remained no change after ADAM9 silencing. IHC staining of MICA also indicated that there was no difference between TNBC metastasis group and TNBC non-metastasis group (data not shown). Wang et al. reported that in MDA-MB-231 cells, the siNSD2-induced inhibition of NSD2 was associated with decreased expression of ADAM9 and EGFR, and further led to decreased level of p-EGFR. However, they did not investigate whether the reduced level of p-EGFR was related to the inhibition of ADAM9 (35). In our present study, we investigated whether ADAM9 expression was associated with the phosphorylation of EGFR in TNBC cells. We found that either EGFR or p-EGFR expression did not change after ADAM9 silencing. In addition, we found that the level of HB-EGF was not changed in culture medium supernatant of MDA-MB-231 cells after ADAM9 silencing. It is indicated that ADAM9 is not involved in the phosphorylation of EGFR in TNBC cells.

The activation of NF-κB complex is mediated by AKT activity (36), and NF-κB complex plays a critical role in regulating cell apoptosis and the expression of matrix metalloproteases (MMPs) (37). Aberrant NF-κB complex activation in cancer cells could inhibit the cell apoptosis and increase the expression of MMPs to activate metastasis (38). Several studies showed that inhibition of NF-κB reduced breast cancer progression (39, 40). Though our results showed that inhibited ADAM9 expression had no effect on MDA-MB-231 cells apoptosis (data not shown), ADAM9 silencing suppressed the activation of NF-κB and IκBα in our study. As a transcription factor, NF-κB regulates the target gene expression and transcription by translocating from cytoplasm into nucleus. MMP9 and MMP2, proteins involved in degrading extracellular matrix to promote migration and invasion, are the predominant target gene of NF-κB (41). Overactivated NF-κB signaling upregulates MMP9 and MMP2 expression to facilitate migration and invasion of cancer cells (42, 43). Increased ADAM9 mRNA expression is correlated with increased MMP9 secretion by interaction with β1 integrins to modulate MMP9 synthesis (44). In HCC, ADAM9 mRNA expression is highly associated with MMP2 activity (19). Consistent with these findings, we observed a trend that increased ADAM9 mRNA level was associated with enhanced MMP9 and MMP2 mRNA level in patients with TNBC from TCGA database. However, the mechanism that ADAM9 activates AKT pathway is still unclear. We speculated that overexpressed ADAM9 may activate AKT and NF-κB through cross-talking with integrins. Integrins are a group of transmembrane proteins which involve in cell-extracellular matrix adhesion by activating signal transduction pathway. Integrin αvβ3 was overexpressed in two TNBC cell lines (MDA-MB-231 and MDA-MB-435) compared with Non-TNBC cell lines (T47D and MCF-7 with fgf-4 and lacZ) (45). The expression level of α6 integrin subunit was overexpressed in TNBC cell lines (MDA-MB-231 and MDA-MB-435) (46). Activation of integrins enhanced PI3K signaling and promoted invasion of breast tumor (47). Specifically, Integrin plays a mechanotransducted role in PI3K-induced breast cancer invasion (48). The cluster of αvβ3 integrin triggers activation of PI3K/AKT and NF-κB pathway in MDA-MB-231 cells (49). ADAM9, a member of the metalloprotease-disintegrin family, could bind to several integrins (α6β1 and αvβ5) to regulate cell adhesion and migration (6, 7). In our study, we observed decreased levels of p-AKT and p-NF-κB with unchanged level of HB-EGF and p-EGFR under ADAM9 silencing in TNBC cells. Gathering with results of above findings, we plausibly speculate that ADAM9 binds to integrins to triggers the activation of PI3K/AKT and NF-κB in TNBC cells. However, we could not exclude the possibility that ADAM9 might indirectly regulate NF-κB pathway due to the proteinase activity of MMP9 and MMP2. Many factors, such as TGF-β, activating protein-1 (AP-1) and EGF, interact with the promoter of MMP9 and MMP2 to induce the expression of MMP9 and MMP2 in cancer cells (50). Overexpressed MMP9 and MMP2 involve in degradation of extracellular matrix and cleavage of integrins to induce the invasion and tumorigenesis (51), which might activates NF-κB pathway. More details are required to demonstrate whether ADAM9 silencing could influence the expression of MMP9 and MMP2 *in vitro* and *in vivo*. Furthermore, the role of ADAM9 in promoting TNBC progression should be demonstrated in animal model.

In conclusion, ADAM9 is overexpressed in TNBC cells and patients. The current study demonstrates that ADAM9 is an important molecule in mediating TNBC aggressiveness and may be a potential useful therapeutic target in TNBC treatment.

## Data Availability Statement

All datasets generated for this study are included in the article/Supplementary Material.

## Ethics Statement

The study involving human samples were reviewed and approved by to the Research Ethics Committee of the Kowloon Central/Kowloon East Cluster under the Hospital Authority (Ref: KC/KE-19-0114/ER-2).

## Author Contributions

LC, HZ, MT, and WCSC initiated the project and designed the experiments. RZ performed the experiments and data analysis. VM and WCSC performed the IHC assay and analysis. WC and Y-KS collected clinical samples and pathological input. RZ finished the manuscript writing. All authors were involved in discussion and revising of the manuscript.

## Conflict of Interest

The authors declare that the research was conducted in the absence of any commercial or financial relationships that could be construed as a potential conflict of interest.

## Supplementary Material

Supplementary Figure 1 The proliferation **(A)** and migration **(B)** of MCF-7 were not affected by ADAM9 silencing.

Supplementary Figure 2 Positive spearman correlation between ADAM9 mRNA levels and MMP9 mRNA levels **(A)** and MMP2 **(B)** in TNBC patients from TCGA database (*n* = 116).
