# Supplementary figures and images for: ADAM9 Mediates Triple-Negative Breast Cancer Progression via AKT/NF-κB Pathway
Source: Front Med (Lausanne). 2020 Jun 19;7:214. doi: 10.3389/fmed.2020.00214 (PMC7317048; doi:10.3389/fmed.2020.00214)

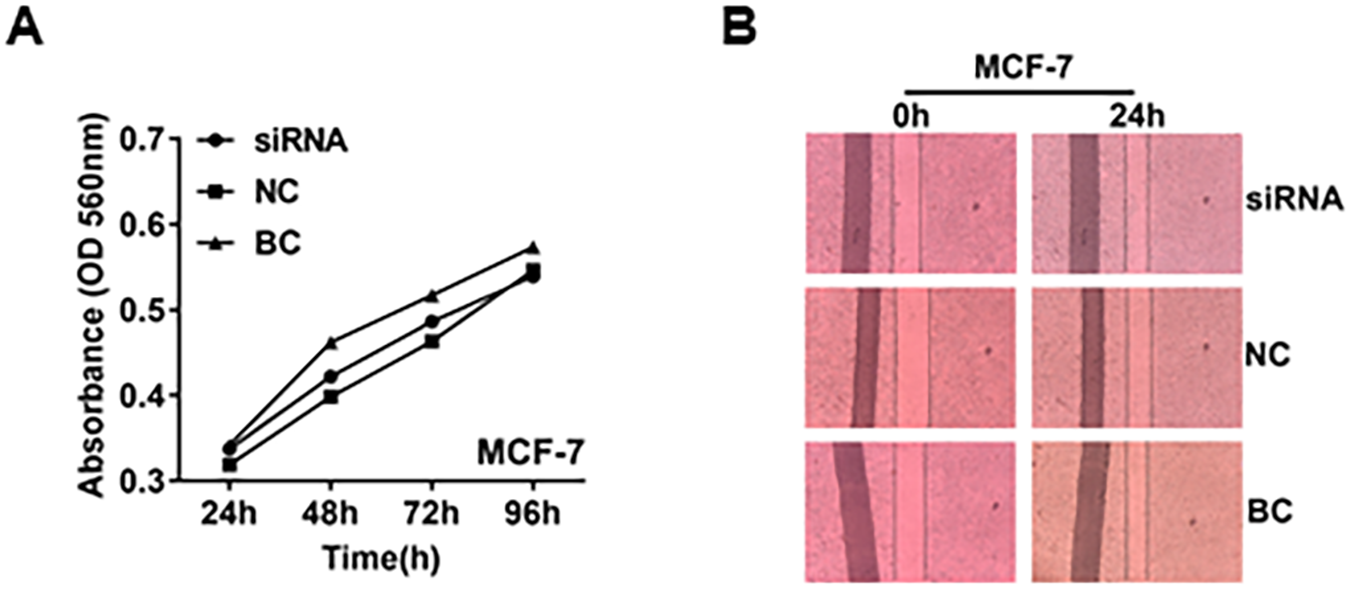

Supplement: Supplementary Figure 1 — The proliferation (A) and migration (B) of MCF-7 were not affected by ADAM9 silencing. [file Image_1.tif]

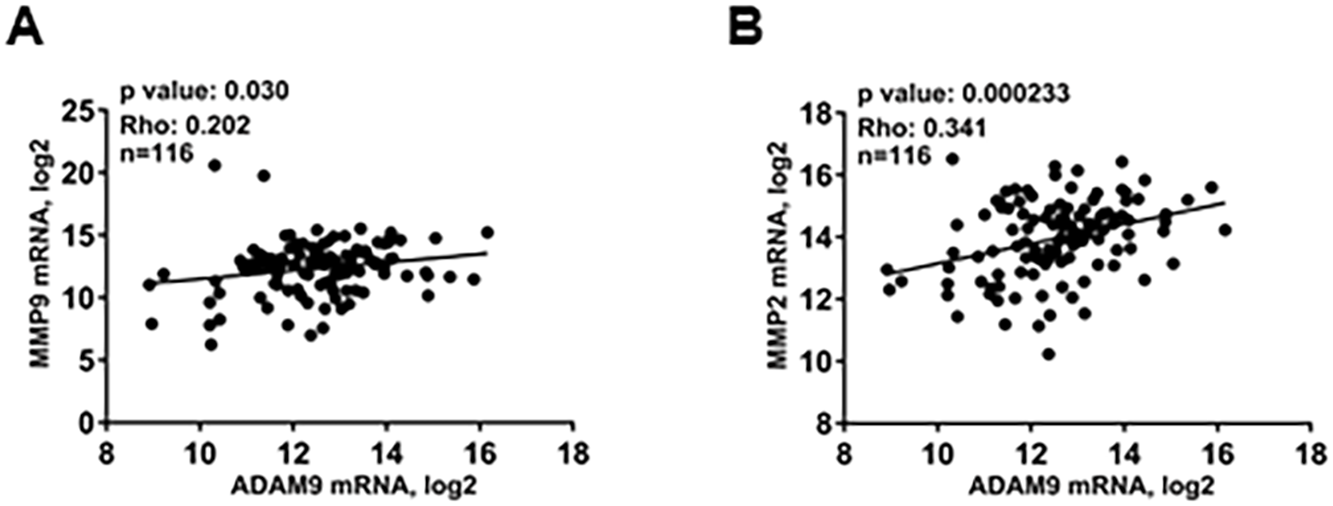

Supplement: Supplementary Figure 2 — Positive spearman correlation between ADAM9 mRNA levels and MMP9 mRNA levels (A) and MMP2 (B) in TNBC patients from TCGA database (n = 116). [file Image_2.tif]
